# TREK channel activation suppresses migraine pain phenotype

**DOI:** 10.1016/j.isci.2021.102961

**Published:** 2021-08-08

**Authors:** Pablo Ávalos Prado, Arnaud Landra-Willm, Clément Verkest, Aurore Ribera, Anne-Amandine Chassot, Anne Baron, Guillaume Sandoz

**Affiliations:** 1 Université Côte d’Azur, CNRS, INSERM, iBV, Nice, France; 2 Laboratories of Excellence, Ion Channel Science and Therapeutics, Nice, France; 3 Fédération Hospitalo-Universitaire InovPain, Cote d'Azur University, University Hospital Centre Nice, Nice, Provence-Alpes-Côte d'Azur, France; 4 Universite Côte d’Azur, CNRS, Institut de Pharmacologie Moléculaire et Cellulaire, Valbonne, France

**Keywords:** pharmacology, biological sciences, neuroscience, behavioral neuroscience, Sensory neuroscience

## Abstract

Activation and sensitization of trigeminal ganglia (TG) sensory neurons, leading to the release of pro-inflammatory peptides such as calcitonin gene-related peptide (CGRP), are likely a key component in migraine-related headache induction. Reducing TG neuron excitability represents therefore an attractive alternative strategy to relieve migraine pain. Here by using pharmacology and genetic invalidation *ex vivo* and *in vivo*, we demonstrate that activating TREK1 and TREK2 two-pore-domain potassium (K_2P_) channels inhibits TG neuronal firing sufficiently to fully reverse the migraine-like phenotype induced by NO-donors in rodents. Finally, targeting TREK is as efficient as treatment with CGRP antagonists, which represents one of the most effective migraine therapies. Altogether, our results demonstrate that inhibiting TG excitability by pharmacological activation of TREK channels should be considered as an alternative to the current migraine treatment.

## Highlights

• *Trek1* and *Trek2* invalidation increases TG excitability and generates migraine
• TREK1 and TREK2 activation prevents migraine in rodents
• TREK1/TREK2 agonists are as good as the most efficient current cares for migraine
• Targeting TREK channels needs to be considered for clinical treatment on migraine

## Graphical abstract

**Figure:**
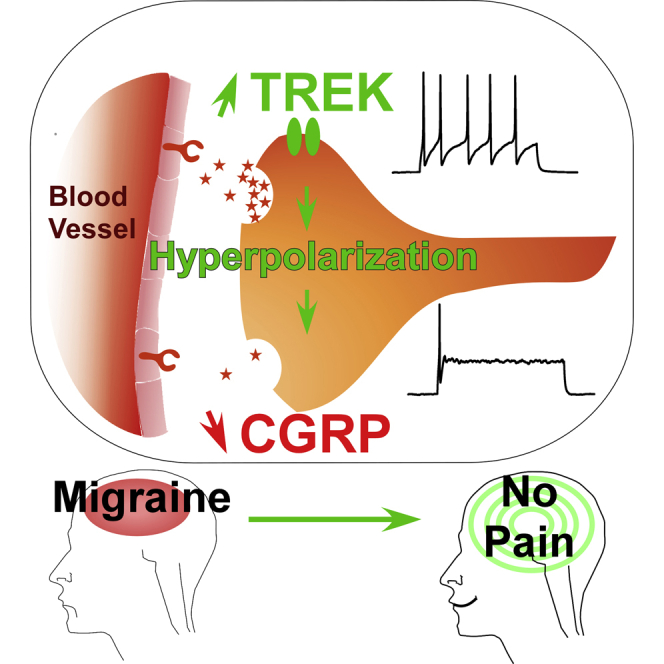

## Introduction

Migraine is considered one of the most disabling conditions worldwide, affecting around 15% of the global population (Burch et al., 2015). It manifests as a unilateral throbbing headache and is accompanied by multiple symptoms such as nausea, vomiting, photophobia, phonophobia, or cutaneous allodynia. It is generally assumed that migraine attacks start with the activation of sensory neurons in the trigeminal ganglia (TG) (Strassman et al., 1996). Hyperexcitability of TG fibers innervating meninges induces the release of neuropeptides (*e.g.* substance P and the calcitonin gene-related peptide [CGRP]) that triggers vasodilatation of surrounding blood vessels and local neurogenic inflammation, leading to migraine (Frederiksen et al., 2019; Goadsby et al., 2017; Khan et al., 2019). Targeting the pro-inflammatory peptides secreted by TG neurons such as CGRP constitutes one of the most effective current strategy to treat migraine (Edvinsson et al., 2018). However, about 50% of the patients do not respond to this treatment and suffer from secondary effects (joint pain, dizziness, constipation, flu-like symptoms) and alternative patient care needs to be developed.

Expression of several K_2P_ channel subunits has been detected in nociceptive dorsal root ganglions and trigeminal neurons (Alloui et al., 2006; Yamamoto et al., 2009)*.* We recently found that the human mutation of K_2P_-TRESK channel, “TRESK-MT”, related to migraine, inhibits K_2P_-TREK1 and TREK2 channels and sufficiently increases sensory TG neuronal excitability to generate a migraine-like phenotype (Royal et al., 2019). These results demonstrate the importance of TREK1 and TREK2 channels in the regulation of TG excitability.

As the current most effective drugs against migraine target the neuropeptide CGRP released by TG neurons or its receptor (Edvinsson et al., 2018), it is attractive to act on TG excitability to prevent CGRP release and, consequently, migraine. We therefore made the hypothesis that enhancing TREK1 and TREK2 activity would reduce TG excitability, prevent the release of pro-inflammatory peptides, and therefore suppress migraine pain. We show that, at a cellular level, ML67-33, a TREK1/2 agonist (Bagriantsev et al., 2013) reduces TG sensory neuronal excitability, explaining its “anti-migraine” properties. Using behavioral tests in wild-type and double knock-out mice for *Trek1/Trek2* (*Trek1*^*−/−*^*-Trek2*^*−/−*^ mice)*,* we demonstrate that ML67-33 reverses the NO donor-induced migraine-like phenotype. This reversion exhibits a similar potency to BIBN4096, a selective CGRP antagonist used against migraine (Olesen et al., 2004). Finally, we demonstrate that ML67-33 completely reversed the NO donor-induced facial allodynia in rats, which is a direct index of TG excitability. Therefore, targeting TREK channels to inhibit TG firing and prevent CGRP release underlying migraine should therefore be considered when developing molecules to treat migraine.

## Results

### TREK1 and TREK2 activation by ML67-33 reverses the migraine-like cutaneous allodynia in mice

To investigate whether targeting TREK1 and TREK2 activity would treat migraine, we first verified whether the TREK agonist ML67-33 (Bagriantsev et al., 2013) specifically activated TREK1 and TREK2 channels. We validated that ML67-33 selectively activated both TREK1 and TREK2 homodimer, as well as TRESK-TREK1 heterodimer currents, whereas TRESK alone was not activated by ML67-33 (Figure S1). This shows that ML67-33, by binding to only one channel moiety, can agonize the TREK channels, making it the molecule of choice for studying TREK1/2 homo- and heterodimer channel function.

**Figure 1:**
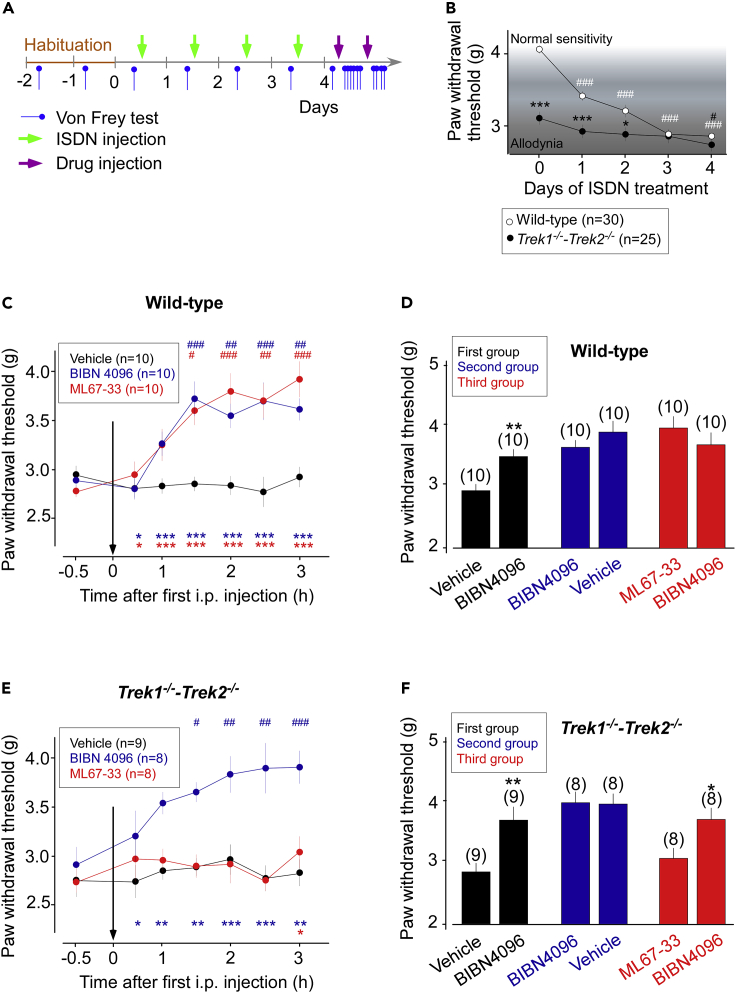
ML67-33 reverses NO-induced migraine-like-phenotype through TREK channel activation (A) Migraine behavioral test timetable. (B) Effect of repeated administration of systemic ISDN NO-donors (10 mg/kg i.p.) on the mechanical threshold (g) and the development of cutaneous allodynia related to migraine in wild-type and *Trek1*^*−/−*^*-Trek2*^*−/−*^ mice. Two-way ANOVA and Sidak post hoc vs wild-type (∗p < 0.5, ∗∗∗p < 0.001); One-way ANOVA and Dunett's post hoc vs pre-treatment day 1 (#p < 0.5, ###p < 0.001). (C) Time course of cutaneous mechanical threshold (g) after injection of a vehicle solution (saline + DMSO 0.1% i.p.), BIBN4096 (1 mg/kg i.p.) and ML67-33 (1 mg/kg i.p.) in wild-type mice. Two-way ANOVA and Dunett's post hoc vs vehicle (∗p < 0.5, ∗∗p < 0.01, ∗∗∗p < 0.005); One-way ANOVA and Dunett's post hoc vs time −0.5 h (#p < 0.5, ##p < 0.01, ###p < 0.005). (D) Bar graph representing mechanical threshold (g) of the three groups of animals in this study after 2h of the second injection of BIBN4096, vehicle and BIBN4096 respectively. Student's t-test (∗p < 0.5, ∗∗p < 0.01). (E and F) Same as C-D for *Trek1*^*−/−*^*-Trek2*^*−/−*^ mice.

Once validated, we next monitored the effects of ML67-33 in a well-described model of chronic migraine in mice induced by nitric oxide (NO) donors (Bates et al., 2010) such as isosorbide dinitrate (ISDN) (Dallel et al., 2018; Royal et al., 2019; Verkest et al., 2018). We induced and evaluated cutaneous allodynia, a quantifiable marker of migraine, after four consecutive days of ISDN injections in wild-type and *Trek1*^*−/−*^*-Trek2*^*−/−*^ mice by measuring paw withdrawal mechanical threshold using a dynamic von Frey esthesiometer before each injection (Figure 1A). We first measured the consequences of ISDN injection on the mechanical threshold. In wild-type animals, we observed a significant decrease of this threshold when we compared the first and last measurements (4.04 ± 0.06 g vs 2.87 ± 0.04 g, p < 0.001). *Trek1*^*−/−*^*-Trek2*^*−/−*^ mice already exhibited a low mechanical threshold, as previously reported (Royal et al., 2019) (4.04 ± 0.06 g vs 3.11 ± 0.08 g for wild-type and *Trek1*^*−/−*^*-Trek2*^*−/−*^ mice before ISDN i.p., respectively, p > 0.001) (Figure 1B).

We then compared the effect of ML67-33 with the effect of BIBN4096, a CGRP antagonist used for migraine treatment (Olesen et al., 2004), and challenged the capacity of ML67-33 to increase the lowered mechanical threshold induced by ISDN injections. For this purpose, wild-type or *Trek1*^*−/−*^*-Trek2*^*−/−*^ mice were classified into three groups (for each), following a series of two different injections spaced four hours in time (Figures 1D and 1F). The first group was first injected with a vehicle solution (saline + DMSO 0.1%) then with BIBN4096. The second group was first injected with BIBN4096 then with the vehicle solution. The third group was first injected with ML67-33 and then BIBN4096.

The efficacy of BIBN4096 was confirmed in both wild-type and *Trek1*^*−/−*^*-Trek2*^*−/−*^ mice from the first group, since the second injection with BIBN4096 strongly increased their mechanical threshold compared with the first injection with the vehicle solution (2.92 ± 0.10 g vs 3.47 ± 0.11 g and 2.79 ± 0.13 g vs 3.28 ± 0.26 g, for wild-type and *Trek1*^*−/−*^*-Trek2*^*−/−*^ mice, respectively, p > 0.2 for both conditions) (Figures 1D and 1F, black graphs). In the second group, the mechanical threshold rapidly increased in both wild-type and *Trek1*^*−/−*^*-Trek2*^*−/−*^ animals after the first injection with BIBN4096 (2.89 ± 0.07 g vs 3.61 ± 0.11 g, p < 0.01; and 2.87 ± 0.18 g vs 3.87 ± 0.17 g, p < 0.001, for wild-type and *Trek1*^*−/−*^*-Trek2*^*−/−*^ mice, respectively) (Figures 1C and 1E, blue traces), and exhibited no additive effects following the second injection with the vehicle solution (3.61 ± 0.11 g vs 3.85 ± 0.17 g and 3.87 ± 0.17 g vs 3.82 ± 0.18 g, for wild-type and *Trek1*^*−/−*^*-Trek2*^*−/−*^ mice, respectively) (Figures 1D and 1F, blue graphs). Nevertheless, wild-type and *Trek1*^*−/−*^*-Trek2*^*−/−*^ mice from the third group responded differently, whereas the first injection of ML67-33 increased the mechanical threshold of wild-type animals compared with the control (2.78 ± 0.06 g vs 3.92 ± 0.18, p < 0.001) (Figure 1C, red trace), and it had no significant impact on *Trek1*^*−/−*^*-Trek2*^*−/−*^ mice (2.69 ± 0.11 g vs 3.00 ± 0.16, p > 0.1) (Figure 1E, red trace). Interestingly, the second injection of BIBN67-33 had no additive effect on the threshold in wild-type mice after the first injection (3.92 ± 0.18 g vs 3.65 ± 0.19, p > 0.1) (Figure 1D, red graph), while it increased the threshold in *Trek1*^*−/−*^*-Trek2*^*−/−*^ mice (3.00 ± 0.16 g vs 3.61 ± 0.18, p < 0.05) (Figure 1F, red graph).

Together, these results indicate that either antagonizing CGRP receptors using BIBN4098 or specifically activating TREK1/2 using ML67-33 similarly reverses from NO-donors-induced allodynia related to migraine in mice.

### ML67-33 increases TREK1 and TREK2 currents in TG sensory neurons

To investigate whether the ML67-33 agonist is specifically regulating TREK1/2 currents in sensory neurons to reduce allodynia, we studied its effects on small and medium-sized TG sensory neurons either from wild-type or double KO animals. These neurons are classically divided within two populations, according to their ability to bind the plant lectin isolectin B4 (IB4) from *Griffonia simplicifolia* (Stucky and Lewin, 1999). Both IB4^+^ and IB4^-^ neurons are involved in nociception but only IB4^-^ are enriched in neuropeptides, notably CGRP, known for inducing neurogenic inflammation and triggering migraine attacks (Frederiksen et al., 2019; Khan et al., 2019).

**Figure 2:**
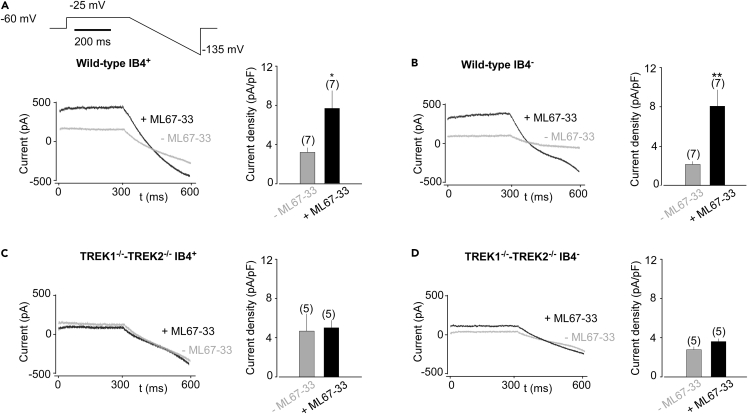
ML67-33 potentiates TREK channels in TG neurons (A–D) Representative current traces of IB4^+^ and IB4^-^ neurons of small TG neurons obtained from wild-type (A and B) and *Trek1*^*−/−*^*-Trek2*^*−/−*^ (C and D) mice, before and during perfusion of ML67-33 (10 μM). Insets: Bar graphs representing current densities at −25 mV (pA/pF). Currents were elicited by voltage-ramps (from −25 to −135 mV, 300 ms duration). Paired t test (∗p < 0.05, ∗∗p < 0.01). Mean ± SEM.

On primary cultured TG neurons from wild-type mice, perfusion of ML67-33 increased the amplitude of a sustained K^+^ current in IB4^+^ and IB4^-^ neurons (3.18 ± 0.46 pA/pF and 7.66 ± 1.80 and pA/pF before and after exposure to ML67-33, respectively for IB4^+^ neurons, p < 0.05; 2.19 ± 0.30 pA/pF and 8.12 ± 1.62 pA/pF before and after exposure to ML67-33, respectively for IB4^-^ neurons, p < 0.01, at −25 mV) (Figures 2A and 2B). However, no significant difference was detected in the currents obtained from *Trek1*^*−/−*^*-Trek2*^*−/−*^ TG neurons before and after treatment with the TREK agonist (4.71 ± 1.75 pA/pF and 5.05 ± 0.76 and pA/pF before and after exposure to ML67-33, respectively for IB4^+^, p > 0.3; 2.77 ± 0.31 pA/pF and 3.60 ± 0.29 pA/pF before and after exposure to ML67-33, respectively for IB4^-^, p > 0.05, at −25 mV) (Figures 2C and 2D). Therefore, ML67-33 specifically activates TREK1 and/or TREK2 currents in TG sensory neurons.

### TREK current activation reduces intrinsic excitability in TG neurons related to migraine

**Figure 3:**
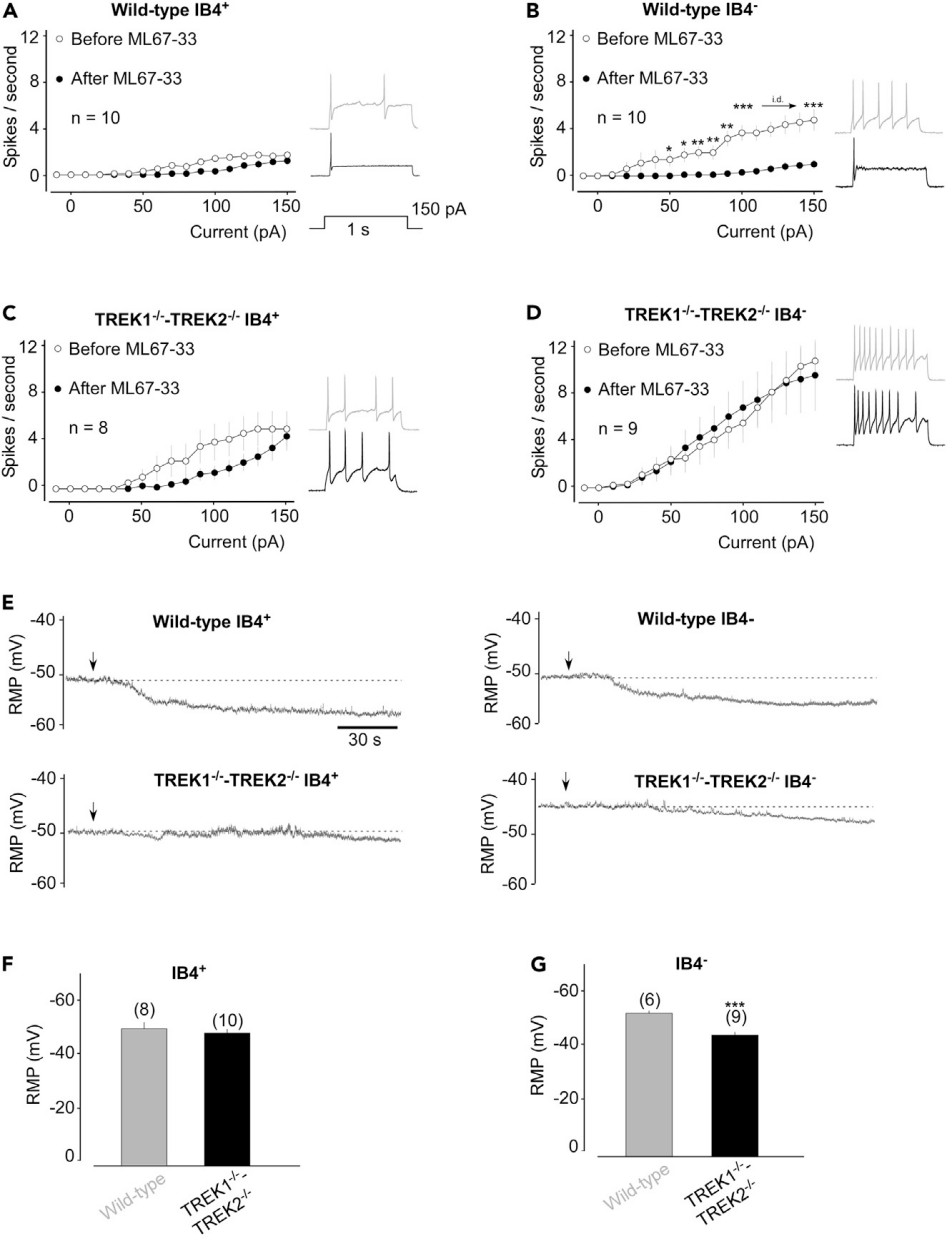
ML67-33 reduces TG neuron excitability through TREK channels activation (A–D) Input-output plots of spike frequency in response to 1-s depolarizing current injection in small IB4^+^ and IB4^-^ small TG neurons from wild-type (A and B) and *Trek1*^*−/−*^*-Trek2*^*−/−*^ (C and D) mice before and after perfusion with ML67-33 (10 μM). At right, Representative traces of action potentials generated by incremental depolarizing current injections in small-diameter TG neurons. Two-way ANOVA (∗p < 0.5, ∗∗p < 0.01, ∗∗∗p < 0.001). (E) Representative RMP (mV) recordings from TG neurons exposed to ML67-33. (F and G) Bar graphs showing the basal RMP value of IB4^+^ (F) and IB4^-^ (G) TG neurons. Mann-Whitney test (∗∗∗p < 0.001). Mean ± SEM.

We next determined the functional consequences of the TREK1-TREK2 current on TG sensory neuron excitability. First, our results showed that *Trek1* and *Trek2* genetic deletion increased both IB4^+^ and IB4^-^ neurons excitability (Figure 3 A-D, white points and gray traces; 1.70 ± 0.33 Hz vs 5.12 ± 1.52 Hz for IB4^+^ from wild-type and *Trek1*^*−/−*^*-Trek2*^*−/−*^, respectively, p < 0.05; 5.12 ± 1.52 Hz vs 10.89 ± 1.57 Hz for IB4^-^ from wild-type and *Trek1*^*−/−*^*-Trek2*^*−/−*^ respectively, p < 0.01, for a 150 pA current injection stimulus), indicating that TREK1/2 activation is required to reduce sensory neuron excitability.

Second, in wild-type IB4^+^ and IB4^-^ sensory neurons, the TREK current increased by ML67-33 led to a non-significant decrease of the spike frequency for IB4^+^ neurons (1.70 ± 0.33 Hz vs 1.20 ± 0.36 Hz, p > 0.2) (Figure 3A) and an important reduction of the spike frequency in IB4^-^ neurons (4.80 ± 0.90 Hz vs 1.00 ± 0.36 Hz, p > 0.001) (Figure 3B). The ML67-33-induced firing inhibition might not be detected in IB4^+^ neurons because at rest their firing rate is already low (Guo and Cao, 2014; Guo et al., 2014). The reduction of the IB4^-^ excitability is specific to the activation of TREK channels since ML67-33 was not effective in sensory neurons from *Trek1*^*−/−*^*-Trek2*^*−/−*^ (IB4^+^: 5.12 ± 1.51 Hz vs 4.50 ± 1.19 Hz before and after ML67-33 perfusion, respectively, p > 0.3; IB4^-^: 10.89 ± 1.57 Hz and 9.67 ± 3.03 Hz before and after ML67-33 perfusion, respectively; p > 0.6) (Figures 3C and 3D). Furthermore, the rheobase (*i.e.* the lowest current intensity necessary for triggering action potentials (APs) was significantly enhanced by ML67-33 in wild-type sensory neurons only (Figure S2). Together, this indicates that activating TREK1/2 using ML67-33 specifically reduces IB4^-^ neuronal excitability.

Excitability is closely linked to the resting membrane potential (RMP). Because K_2P_ channels serve as a hub for RMP maintenance at negative values (Enyedi and Czirják, 2010), we evaluated the effect of ML67-33 on this parameter. We found that ML67-33 induced hyperpolarization of TG neurons from wild-type animals (Figures 3E and S3, −51.32 ± 2.40 mV and −62.13 ± 2.84 mV, before and after perfusion of ML67-33, respectively for IB4^+^, p < 0.001; −53.96 ± 0.91 mV and −61.46 ± 2.21 mV, before and after perfusion of ML67-33, respectively for IB4^-^, p < 0.01) mainly through TREK1 and TREK2 activation since the slight RMP decrease observed for *Trek1*^*−/−*^*-Trek2*^*−/−*^ TG neurons was not statistically significant (−49.94 ± 1.42 mV and −53.94 ± 2.28 mV before and after perfusion of ML67-33, respectively, for IB4^+^, p > 0.2; −45.70 ± 1.12 mV and −50.70 ± 1.81 mV before and after perfusion of ML67-33, respectively, for IB4^-^, p > 0.06). In addition, we noticed that the basal value of the RMP of peptidergic neurons from *Trek1*^*−/−*^*-Trek2*^*−/−*^ animals was statistically higher than those from wild-type mice (−45.70 ± 1.12 mV vs −53.96 ± 0.91 mV for *Trek1*^*−/−*^*-Trek2*^*−/−*^ and wild-type peptidergic neurons, respectively, p < 0.001) (Figure 3G). These results confirm that, at rest, TREK1 and TREK2 control the RMP basal value.

Altogether, these data show that TREK1 and TREK2 play a crucial role in the excitability of TG neurons, and particularly of peptidergic IB4^-^ nociceptive neurons, by determining the threshold for the generation of APs and the spike frequency and by notably setting up the RMP at values close to the K^+^ equilibrium potential. Thus, ML67-33 increases TREK current to reduce the TG excitability, leading to a decrease of the migraine phenotype observed in wild-type animals treated with NO-donors.

### Activation of TREK1 and TREK2 abrogates migraine-like facial allodynia in rats

**Figure 4:**
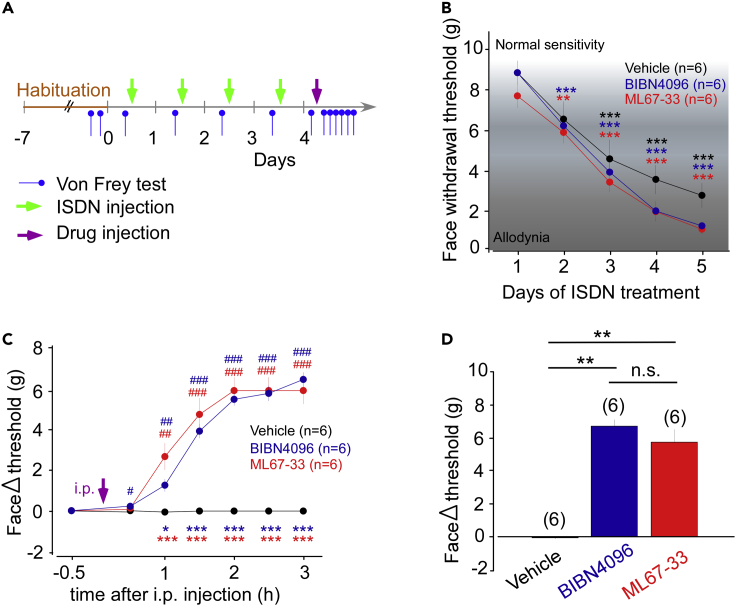
ML67-33 abrogates mechanical facial allodynia related to migraine (A) Migraine behavioral test timetable. Green arrows represent the injection of ISDN, a known migraine trigger. Magenta arrows represent the injection of either saline or BIBN4096 or ML67-33 solution injection. Blue arrows represent the measurement of the facial withdrawal mechanical threshold. (B) Effect of repeated administration of systemic ISDN (10 mg/kg i.p.) on the occurrence and development of cutaneous chronic allodynia (facial mechanical threshold, g) in rats before treatment with different drugs. One-way ANOVA and Sidak post hoc vs pre-treatment day 1 (∗∗p < 0.01, ∗∗∗p < 0.001). (C) ML67-33 is as efficient as the CGRP receptor antagonist BIBN4096 to reverse migraine like phenotype: time-evolution of von Frey delta facial withdrawal thresholds in rats injected with a vehicle solution (saline + DMSO 0.1% i.p.), BIBN4096 (1 mg/kg i.p.) and ML67-33 (1 mg/kg i.p.). Two-way ANOVA and Sidak post hoc vs vehicle treatment (∗p < 0.05, ∗∗∗p < 0.001); One-way ANOVA and Dunett's post hoc vs time - 0.5 h (#p < 0.5, ##p < 0.01, ###p < 0.001). (D) Variation of the face withdrawal threshold before and 3 hours after compound administration. Kruskal-Wallis and Student-Newman-Keuls post hoc (∗∗p < 0.01). Mean ± SEM.

To validate that injection of ML67-33 decreases TG sensory neuronal excitability and suppresses NO-migraine phenotype *in vivo*, we conducted behavioral experiments in rats. This model allows to measure the mechanical allodynia of the face which is related to TG excitability and therefore constitutes one of the most reliable and quantifiable readout of TG excitability underlying migraine phenotype (Harris et al., 2017; Pradhan et al., 2014; Royal et al., 2019). The protocol used to induce chronic migraine was similar to the one used in mice despite the use of the facial sensitivity instead of the paw withdrawal threshold as a readout. After a week of habituation, the animals were tested for facial mechanical sensitivity with von Frey filaments. Migraine-like phenotype was induced by daily intraperitoneally (i.p.) injections of ISDN (10 mg/kg) for four consecutive days and facial mechanical threshold was measured before each injection over the four days. At day 5, either ML67-33, BIBN4096, or vehicle was administrated and their effects were followed for 3 hours, every 30 min (Figure 4A).

As previously described (Verkest et al., 2018), ISDN induced a strong significant reduction of the face withdrawal threshold compared with normal sensitivity (p < 0.001 for the three groups) (Figure 4B). This reduction of the face withdrawal threshold is associated with the development of chronic mechanical allodynia that reflects the induction of trigeminal hyperexcitability and migraine (Royal et al., 2019). As shown in Figure 4C, both ML67-33 and BIBN4096 treatments increased in a similar manner the mechanical threshold previously lowered by ISDN injections (5.7 ± 0.77 g vs 6.7 ± 0.39 g for rats injected with ML67-33 and BIBN4096, respectively, p > 0.6) (Figures 4C and 4D).

Thus, in rodent models of migraine, activation of TREK1 and TREK2 channels reverses from chronic allodynia related to migraine pain as efficiently as antagonizing CGRP with BIBN4096.

## Discussion

Migraine is a neurological disease caused by the combination of environmental, hormonal, and genetic components. Genome-wide association studies have revealed the importance of genetic mutations in ion channels involved in synaptic transmission in migraine predisposition (Gormley et al., 2016; Nyholt et al., 2008). Particularly, K_2P_ channels such as TRESK were shown to play a fundamental role in this disease. The frameshift mutation TRESK-MT perfectly segregates with migraine phenotype in large pedigree (Andres-Enguix et al., 2012; Lafrenière et al., 2010). The direct causality linking TRESK-MT to migraine was recently demonstrated by showing that using CRISPR-Cas9 to fix the MT mutation restores a normal nociceptor excitability (Pettingill et al., 2019) and that the TRESK-MT mutation generated altered proteins which affect TREK1 and TREK2 channel function. The TRESK-MT fragment binds and inhibits TREK1 and TREK2 in TG sensory neurons, leading to neuronal hyperexcitability and migraine (Royal et al., 2019). This makes TREK1 and TREK2 good candidates for regulating TG excitability and subsequently migraine.

To analyze the potential of modulating TREK channel activity as an alternative target to treat this disease, we combined a pharmacological approach using the TREK agonist ML67-33 with mice models deficient for both *Trek1* and *Trek2*. We found that (i) in wild-type mice, ML67-33 reverses from NO-donors-induced allodynia and is as efficient as the CGRP antagonist BIBN4096, and that (ii) in *Trek1*^*−/−*^*-Trek2*^*−/−*^ mice, ML67-33 has no effect. In addition, despite the fact that TRAAK, a related two-pore-domain potassium (K_2_P) channel, is also modulated by ML67-33 (Bagriantsev et al., 2013), *Trek1*^*−/−*^*-Trek2*^*−/−*^ mice did not restore from ISDN-induce mechanical allodynia, ruling out TRAAK participation in migraine pain. This shows that reduction of allodynia induced by ML67-33 is due to TREK activation. Furthermore, BIBN4096 and ML67-33 do not trigger an additive effect in wild-type mice, while BIBN4096 alone suppresses the allodynia induced by *Trek1/2* genetic deletion. Together, these observations suggest that deleting *Trek1/2* might promote an increase of CGRP release that generates a migraine-like phenotype, whereas their activation might reduce CGRP release and suppresses migraine.

We further confirmed those results by using a rat model and measuring the mechanical threshold on the face, which is directly linked to TG neuronal excitability and represents a good index of migraine state (Harris et al., 2017; Pradhan et al., 2014; Royal et al., 2019). Accordingly, rats injected with NO-donors recovered from induced allodynia after either BIBN4096 or ML67-33 treatment in a similar manner, suggesting that TREK activation may prevent CGRP release to suppress migraine phenotype. At the cellular level, activation of TREK1 and TREK2 dramatically decreased the excitability of TG sensory neurons by notably lowering their RMP value. This reduction of neuronal excitability by ML67-33, only observed in wild-type animals, confirms the role of TREK channels in excitability regulation and consequently in pain transmission.

In conclusion, our results provide evidence of the key role of TREK1 and TREK2 channels in migraine induction by regulating TG excitability, whereas their genetic invalidation induces a neuronal hyperexcitability leading to migraine-like phenotype, their activation suppresses NO-donor induced-migraine phenotype as efficiently as current anti-migraine drugs targeting neuropeptide release. Therefore, targeting TREK channel intrinsic activity to reduce TG neuron excitability should be considered as an alternative strategy to treat migraine.

### Limitations of the study

This study was carried out in rodent models exhibiting an allodynic phenotype which is commonly used as a readout for the migraine-like phenotype studies in rodents. However, other marks such as photophobia may be tested. Furthermore, this study was done in rodents and therefore the results should be confirmed on human patients suffering from migraine.

## STAR★Methods

### Key resources table

**Table:** 

REAGENT or RESOURCE	SOURCE	IDENTIFIER
**Chemicals, peptides, and recombinant proteins**		

BIBN4096 4096	Tocris	Cat# 4561
BSA	Euromedex	Cat# 04-100-812-C
Collagen	This paper	N/A
DMEM	Gibco	Cat# 41965-039
DMEM/F12	Sigma-Aldrich	Cat# 6421
DPBS	Gibco	Cat# 14190-169
FBS	Dutscher	Cat# S1900-500
Glutamine	Gibco	Cat# 25030-081
HEPES	Gibco	Cat# 15630-080
ISDN Risordan ®	Sanofi	N/A
Isolectin GS-IB4	Thermofischer	Cat# I21411
L15 Medium (Leibovitz)	Sigma-Aldrich	Cat# L5520
ML67-33	Tocris	Cat# 6886
Penicillin-Streptomycin	Gibco	Cat# 15140-122
Poly-L-lysine	Sigma-Aldrich	Cat# P4707
Trypsin	Sigma-Aldrich	Cat# T1763

**Deposited data**		

Raw and analyzed data	This paper	https://doi.org/10.17632/4vr86gr5ch.1

**Experimental models: Cell lines**		

HEK 293T cells	ATCC	Cat#CRL11268
Mouse trigeminal ganglia cells	This paper	N/A

**Experimental models: Organisms/strains**		

Mouse: C57BL/6J	Charles River Laboratories	Strain Code 027
Mouse: TREK1^*−/−*^-TREK2^*−/−*^	Guyon et al., 2009	N/A
Rat: Sprague Dawley	Janvier Labs	Strain RjHan:SD

**Oligonucleotides**		

TREK1 (forward): CATCTTCATCCTGTTTGGCTG	Sigma-Aldrich	Custom order
TREK1 (reverse): ATCATGCTCAGAACAGCTGC	Sigma-Aldrich	Custom order
TRESK-TREK1 (internal forward): TTTCGCTACCTTGGGCGGCCCCTGACTTGCTGGAT	Sigma-Aldrich	Custom order
TRESK-TREK2 (internal reverse): GCAAGTCAGGGGCCGCCCAAGGTAGCGAAACTTCC	Sigma-Aldrich	Custom order
TREK2 (forward) AACAGTGGTTGCCATCTTCG	Sigma-Aldrich	Custom order
TREK2 (reverse) CCAGCAAAGAAGAAGGCACT	Sigma-Aldrich	Custom order
TRESK-TREK2 (internal forward): GTTTCGCTACCTTGGAAATTTCCAATCGAGACG	Sigma-Aldrich	Custom order
TRESK-TREK2 (internal reverse): CGTCTCGATTGGAAATTTCCAAGGTAGCAAAC	Sigma-Aldrich	Custom order
TRESK (forward) CTGCTTCCTTTGCTGCCTG	Sigma-Aldrich	Custom order
TRESK (reverse) AAGAAGAGAGCGCTCAGGAA	Sigma-Aldrich	Custom order

**Recombinant DNA**		

pIRES2eGFP	Clontech	6029-1
mTREK1	Royal et al., 2019	N/A
mTREK2	Royal et al., 2019	N/A
mTRESK	Royal et al., 2019	N/A

**Software and algorithms**		

Fiji/ImageJ, v1.8	NIH, Schneider (Schneider et al., 2012) et al., 2012	https://imagej.nih.gov/ij/
pCLAMP 10, pCLAMP 11	Molecular Devices	N/A
SigmaPlot v11	Systat Software Inc.	N/A

**Other**		

Axioplan 2 Imaging Microscope	Zeiss	https://www.micro-shop.zeiss.com/?s=16103145829fcb6&l=en&p=us&f=a&i=10027
Axopatch 200B amplifier	Molecular Devices	N/A
Dynamic plantar aesthesiometer	Ugo Basil	Cat#: 37450
Micromanipulator MP225	Sutter Instrument	https://www.wpi-europe.com/products/micromanipulators/motorised-manipulators/mp-225.aspx
Digidata 1550B	Molecular Devices	N/A
Perfusion	ValveLink 8.2	https://www.autom8.com/perfusion-systems-overview/valvelink8-2-controller/
Camera EMCCD iXon	Andor	https://andor.oxinst.com/products/ixon-emccd-cameras
sCMOS camera Zyla 4.2+	Andor	https://andor.oxinst.com/products/scmos-camera-series/zyla-4-2-scmos
Von Frey Filaments	Bioseb	Model: Bio-VF-M

### Resource availability

#### Lead contact

Further information and requests for resources and reagents should be directed to and will be fulfilled by the lead contact, Guillaume Sandoz (sandoz@unice.fr).

#### Materials availability

This study did not generate new unique reagents and there is no restriction to availability.

### Experimental model and subject details

#### Mice

All mouse experiments were conducted according to national and international guidelines and have been approved by the local ethical committee and authorized the French Ministry of Research according to the European Union regulations and the Directive 2010/63/EU (APAFIS#21943-2019073017246158). The C57BL/6J breeders were maintained on a 12 h light/dark cycle with constant temperature (23–24°C), humidity (45%–50%), and food and water *ad libitum* at the animal facility of Institut de Biologie de Valrose. Knock-out mice lacking *Trek1* and *Trek2* were generated as described (Guyon et al., 2009). Null mutations were backcrossed against the C57BL/6J inbred strain for more than 10 generations prior to establishing the breeding cages to generate subjects for this study. Age- and sex-matched C57BL/6J wild-type mice, aged 9–12 weeks, were obtained from Charles River Laboratories (Wilmington, MA). Behavioral experiments were performed on 7 to 13-weeks old male mice weighting 20–30 g.

#### Rats

Experiments were performed on 6 to 9 weeks old male Sprague-Dawley rats (Janvier Labs) weighing 250 to 400 g. Animals were housed in a 12 hour light-dark cycle with food and water available *ad libitum*. Animal procedures were approved by the Institutional Local Ethical Committee and authorized by the French Ministry of Research according to the European Union regulations and the Directive 2010/63/EU (Agreements 01550.03). Animals were sacrificed at experimental end points by CO_2_ euthanasia.

#### Primary culture of mouse TG neurons

Trigeminal ganglion tissues were collected from postnatal day 2–8 mice of both sex in L15 Leibovitz medium (Sigma) and treated with 2 mg/mL collagenase type II (Worthington) and BSA (Euromedex) for 2 hours, followed by 2.5 mg/mL trypsin (Sigma) for 15 min. Neurons were dissociated in DMEM/F12 medium (Gibco) by triturating with fire-polished and coated glass pipettes and seeded on poly-L-lysine (Sigma) coated coverslips. The DMEM-based culture medium contained 10% fetal bovine serum (Dutscher) and 2 mM Glutamine (Gibco).

#### HEK293T cells

HEK293T (ATCC, #CRL11268) cells were maintained in DMEM (Gibco) supplemented with 10% FBS (Dutscher) in 35 mm dishes. At 70%–80% confluency they were transiently co-transfected using the calcium phosphate method with a total amount of 3.5 μg of DNA and seeded on 18 mm diameter poly-L-lysine (Sigma) coated glass coverslips in 12 well plates.

### Method details

#### Molecular biology and gene expression

Clones used were mTREK1 (GenBank: NM_001159850.1), mTREK2 (GenBank: NM_001316665.1) and mTRESK (GenBank: NM_207261.3). All channel DNA was used in the pIRES2eGFP vector. Heterodimers between TREK1, TREK2 and TRESK were generated by PCR.

#### Electrophysiology

HEK293T electrophysiology was performed 24–48 h after transfection. For whole-cell patch-clamp experiments, cells were recorded in a bath solution containing (in mM) 145 NaCl, 4 KCl, 1 MgCl_2_, 2 CaCl_2_ and 10 HEPES, pH 7.4. The glass pipettes (2–5 MΩ of resistance) were filled with (in mM) 140 KCl, 3 MgCl_2_, 5 EGTA, 10 HEPES, pH 7.3. ML67-33 was perfused at 10 μM for 2–5 min in the bath solution. Whole-cell currents were elicited by voltage-ramps (from −100 to +100 mV, 1 s) holding the cells at −80 mV. Current densities were measured at 0 mV.

For TG neuron electrophysiology, medium and small sized cells (<25 μm) were recorded in an extracellular solution containing (in mM): 135 NaCl, 5 KCl, 2 CaCl_2_, 1 MgCl2, 5 HEPES, 10 glucose, pH 7.4. In order to distinguish between peptidergic and non-peptidergic, neurons were first incubated with an isolectine-IB4 marker (Invitrogen) for 30 min at 5 μg/mL. The pipette solution contained the following (in mM): 140 K-gluconate, 10 NaCl, 2 MgCl_2_, 5 EGTA, 10 HEPES, 2 ATP-Mg, 0.3 GTP-Na, 1 CaCl_2_. Neurons were excluded from analysis when the RMP was higher than −40 mV. To test neuronal excitability, neurons were held at RMP in current-clamp mode and injected with 1 s depolarizing current in 10 pA incremental steps. The voltage-clamp ramp protocol for TREK channels consisted in voltage-ramps from −25 to −135 mV during 600 ms, holding the cells at −60 mV. ML67-33 was perfused at 10 μM for 2–5 min in the extracellular solution.

All cells were recorded at room temperature using an Axopatch 200B (Molecular Devices) amplifier. Signals were filtered at 10 kHz and digitalized at 20 kHz. Cell recordings, data acquisition and analysis of electrophysiology were performed using pClamp software (Molecular Devices).

#### Migraine model rodents

The rodent models of NO-induced migraine were induced by *i.p.* injection of ISDN (Sanofi) at 10 mg/kg, a long-lasting NO donor in both rats and mice.

The mice hindpaw mechanical sensitivity was evaluated with a dynamic plantar aesthesiometer (Ugo Basile). Unrestrained mice were placed in twelve individual plastic boxes on top of a wire surface. The mouse hindpaw was subjected to an increasing force ramp (0–7.5 g in 10 s), the paw withdrawal force threshold (g) was measured in quadriplate on each hind paw, and the mean force was calculated. For 2 days, mice were habituated to repeated (every 30 min) measurements of hindpaw mechanical sensitivities, and basal values were determined 2 days before the experiments. ISDN was injected each day for 4 days, and the mechanical threshold was measured each day before the next ISDN injection to follow the settlement of chronic allodynia. Normal mechanical sensitivity and allodynia thresholds were taken as the measurement values of the first day before treatment with ISDN and the last measurement before the injection of drugs on wild-type mice, respectively. On the fifth day, BIBN4096 (Tocris) (1 mg/kg *i.p*.) and ML67-33 (1 mg/kg, *i.p*.) were administrated as well as their vehicle solution: 0.9% saline and 0.1% DMSO (Sigma). The hindpaw mechanical sensitivity was measured every 30 min before (basal value) and every 30 min after the *i.p*. injections.

The face mechanical sensitivity of rats was measured using calibrated von Frey filaments (Bioseb, France). Unrestrained rats placed in individual plastic boxes on top of a wire surface were trained over 1 week; a stimulus was applied to the periorbital area, following a progressive protocol, starting with non-noxious filaments during the first days of training. The face withdrawal force threshold (g) was determined by the filament evoking at least three responses over five trials, starting with lower force filaments. ISDN (10 mg/Kg, Sanofi) was injected each day for 4 days, and the mechanical threshold was measured each day before the next ISDN injection to follow the settlement of chronic allodynia. To test the effect of drugs, face mechanical sensitivity was measured every 30 min before (basal value) and for 3 h after compound or vehicle injection.

### Quantification and statistical analysis

Analysis of whole-cell currents was performed using ClampFit software. For analysis of voltage-ramp traces cursors were set at 0 mV for HEK293T cells, to extract the average current. Cursors were set at 355 ms for TG neurons. Spike frequency was determined as the number of APs triggered in every current step. The rheobase was taken as the minimal current injected to generate a first AP. When neurons did not trigger any AP even for current injections >500 pA, the rheobase value was considered as 0 pA. Signals were filtered at 10 kHz and digitalized at 20 kHz. For electrophysiology and behavioral experiments, statistical details and n values can be found in the figure legends and in the main text.

## Data Availability

• Source data for the main and supplemental figures in the paper is publicly accessible online at Mendeley https://doi.org/10.17632/4vr86gr5ch.1. • No unpublished custom code, software, or algorithm was used in this study. • Any additional information required to reanalyze the data reported in this paper is available from the lead contact upon request
