# Supplementary material for: TREK channel activation suppresses migraine pain phenotype
Source: iScience. 2021 Aug 8;24(9):102961. doi: 10.1016/j.isci.2021.102961 (PMC8379698; doi:10.1016/j.isci.2021.102961)
Supplement: Document S1. Figures S1–S3 [file mmc1.pdf]

iScience, Volume *24*

Supplemental information

TREK channel activation suppresses migraine pain phenotype

Pablo Ávalos Prado, Arnaud Landra-Willm, Clément Verkest, Aurore Ribera, Anne-Amandine Chassot, Anne Baron, and Guillaume Sandoz

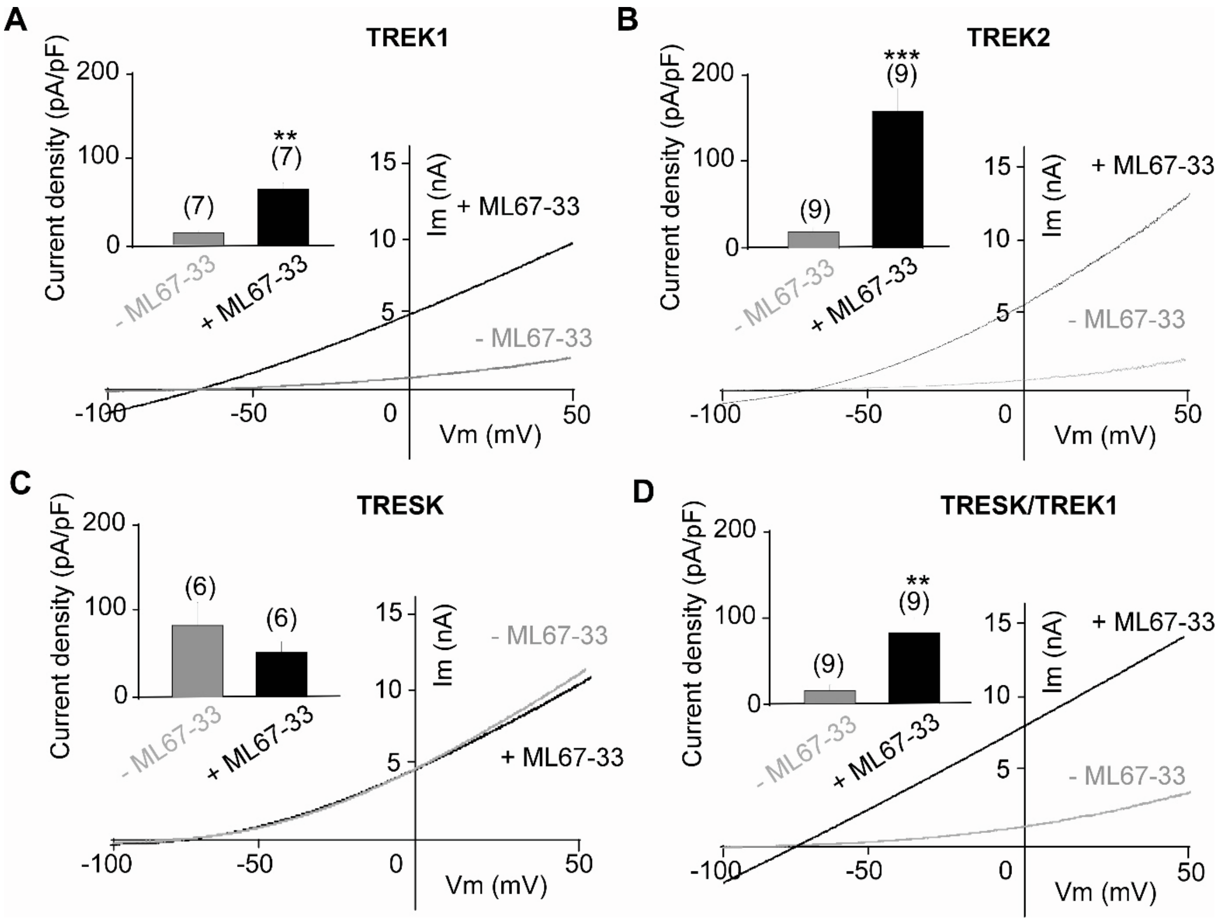

Figure S1. ML67-33 activates TREK1 homodimers and TRESK/TREK1 heterodimers but not TRESK homodimers, Related to Figures 2 and 3. (A-D) Representative current traces obtained from HEK293T cells expressing either TREK1 (A) or TREK2 (B) or TRESK (C) homodimers or TRESK/TREK1 (D) heterodimers before and after application of ML67-33 (10 µM). Currents were elicited by voltage-ramps (from -100 to +100 mV from a holding potential at -80 mV, 1s duration). Insets show a summary of current densities obtained at 0 mV. Paired t-test (** $p < 0.01$; ***$p < 0.001$). Mean ± SEM.

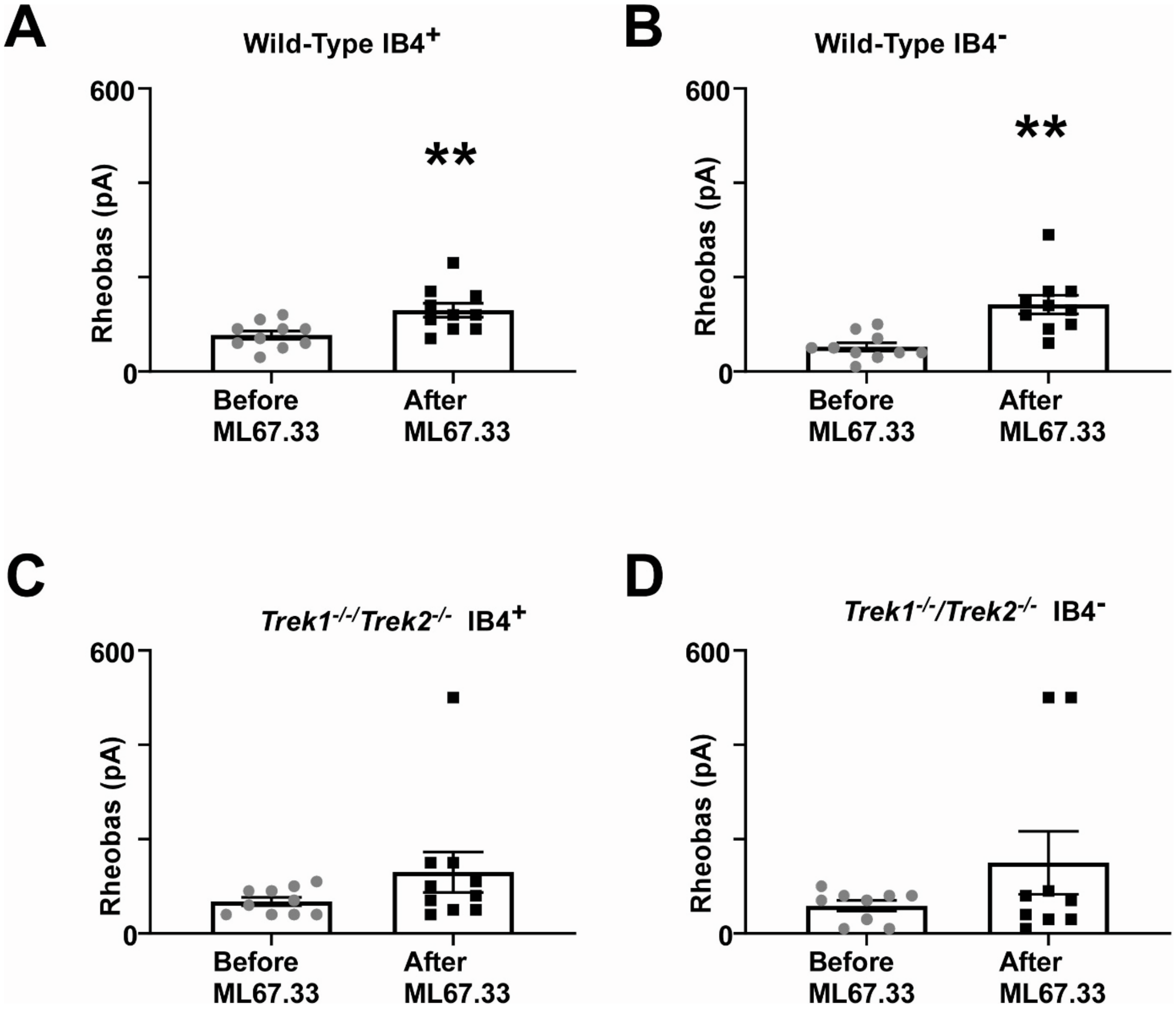

Figure S2. ML67-33 increases rheobase of TG neurons through activation of TREK1 and TREK2 channels, Related to Figure 3. (A-D) Bar graphs showing representing rheobase obtained from $IB4^+$ and $IB4^-$ TG neurons obtained from wild-type (A and B) and *Trek1*$^{-/-}$-*Trek2*$^{-/-}$ (C and D) mice before and after perfusion with ML67-33 (10 µM). Rheobase was determined by incremental depolarizing current injections in steps of + 10 pA in small-diameter TG neurons. Wilcoxon signed-rank test (** $p < 0.01$). Mean ± SEM.

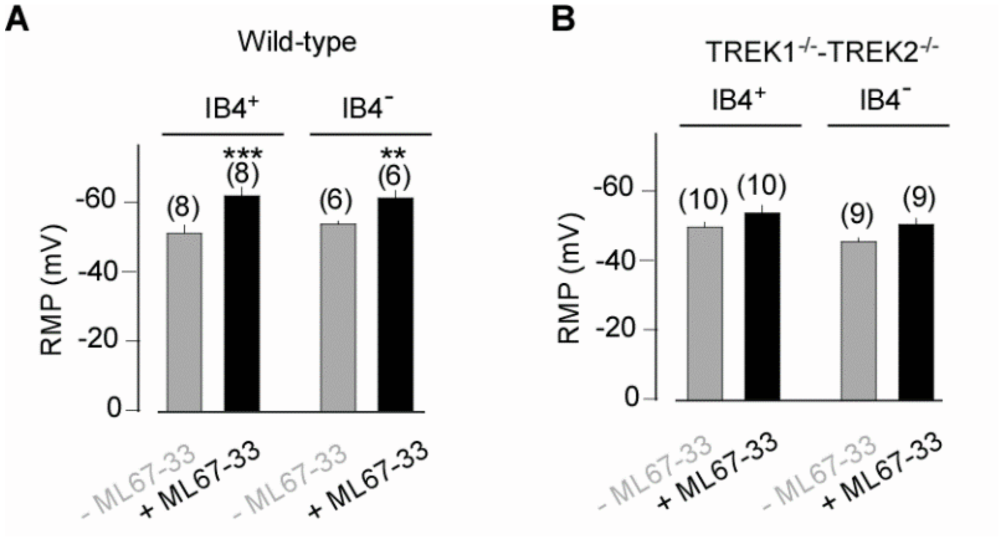

Figure S3. ML67-33 hyperpolarizes TG neurons from wild-type mice through activation of TREK channels, Related to Figure 3. (A-B) Bar graphs showing representing the RMP value obtained from small IB4^{+} and IB4^{-} TG neurons before and after perfusion with ML67-33 (10 µM) from wild-type (A) and *Trek1*$^{-/-}$-*Trek2*$^{-/-}$ (B) mice. Paired t-test (** p < 0.01, ** p < 0.001). Mean ± SEM.
